# Supplementary material for: Learning how to robustly estimate camera pose in endoscopic videos
Source: Int J Comput Assist Radiol Surg. 2023 May 15;18(7):1185–92. doi: 10.1007/s11548-023-02919-w (PMC10329609; doi:10.1007/s11548-023-02919-w)
Supplement: Supplementary file 1 — (pdf 11469 KB) [file 11548_2023_2919_MOESM1_ESM.pdf]

Learning How To Robustly Estimate Camera Pose in Endoscopic Videos

Michel Hayoz[1*], Christopher Hahne[1], Mathias Gallardo[1], Daniel Candinas[2], Thomas Kurmann[3], Maximilian Allan[3] and Raphael Sznitman[1]

[1]ARTORG Center, University of Bern, Switzerland.
[2]Dept. of Visceral Surgery and Medicine, Inselspital, Switzerland.
[3] Applied Research, Intuitive Surgical, USA.

*Corresponding author(s). E-mail(s): michel.hayoz@unibe.ch;

Appendix A StereoMIS dataset

Table A1 Details on StereoMIS dataset. Sequences are denoted x_y where x is the subject and y the sequence number. *length* denotes the total trajectory length of the sequence in millimeters. Number of sequences are reported for the surgical scenarios experiment in the format: total (*breathing*, *scanning*, *deforming*).

	subject	duration	length	fps orig	fps at test-time	split	#sequence
H1_0	human	32min 01s	3516	60.0	30.0	train	-
H2_0	human	3min 10s	116	60.0	30.0	test	7 (5, 1, 1)
H2_1	human	50s	424	60.0	30.0	test	0 (0, 0, 0)
H2_2	human	48s	97	60.0	30.0	test	1 (0, 0, 1)
H3_0	human	5min 19s	781	60.0	30.0	test	6 (5, 1, 0)
P1_0	porcine	5min 19s	1323	60.0	30.0	train	-
P2_0	porcine	3min 28s	434	52.6	26.3	test	8 (4, 4, 0)
P2_1	porcine	2min 36s	1155	54.4	27.2	test	8 (0, 8, 0)
P2_2	porcine	2min 37s	736	54.4	27.2	test	10 (0,10, 0)
P2_3	porcine	2min 35s	302	54.4	27.2	test	4 (0, 4, 0)
P2_4	porcine	2min 37s	1824	54.4	27.2	test	9 (0, 9, 0)
P2_5	porcine	3min 18s	1671	54.2	27.1	test	15 (0,15, 0)
P2_6	porcine	52s	205	26.1	26.1	test	1 (0, 1, 0)
P2_7	porcine	2min 26s	725	37.0	37.0	test	3 (2, 1, 0)
P2_8	porcine	3min 55s	950	39.0	39.0	test	6 (0, 6, 0)
P3_0	porcine	2min 58s	545	60.0	30.0	test	8 (1, 0, 7)

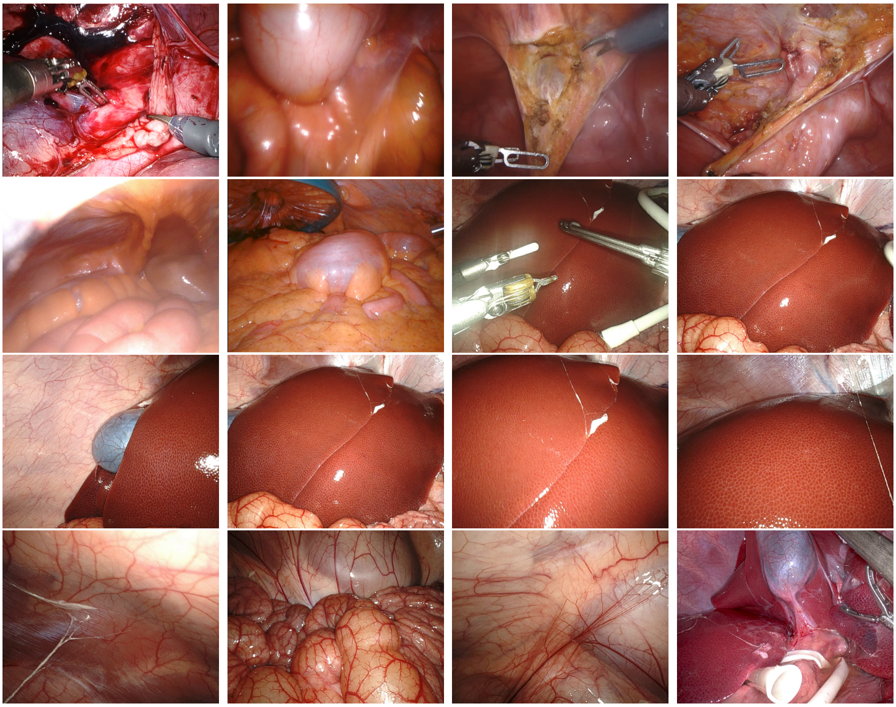

Fig. A1 Example images from StereoMIS sequences. Same order as in table A1 from top left to bottom right.

Appendix B StereoMIS test set trajectories

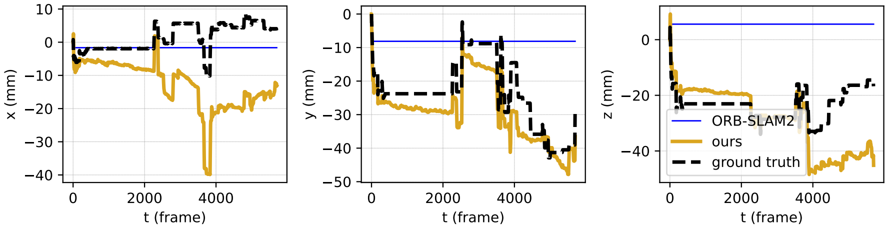

Fig. B2 Trajectory of *StereoMIS* H2_0 (ATE-RMSE 11.3mm).

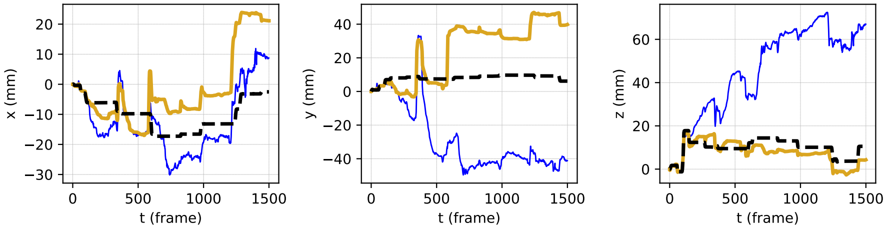

Fig. B3 Trajectory of *StereoMIS* H2_1 (ATE-RMSE 18.8mm).

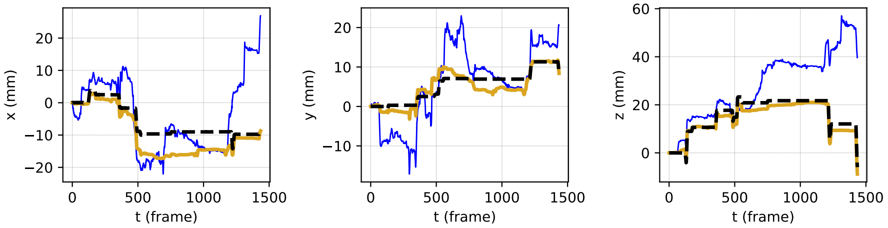

Fig. B4 Trajectory of *StereoMIS* H2_2 (ATE-RMSE 2.6mm).

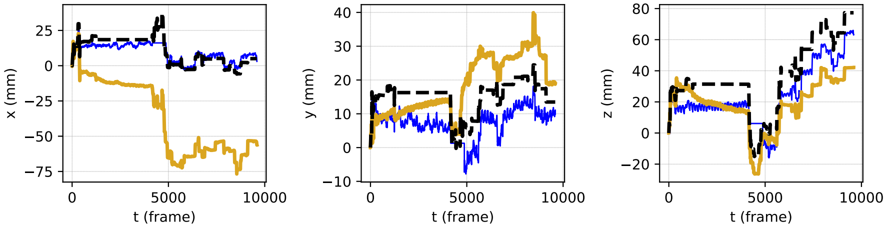

Fig. B5 Trajectory of *StereoMIS* H3_0 (ATE-RMSE 21.2mm).

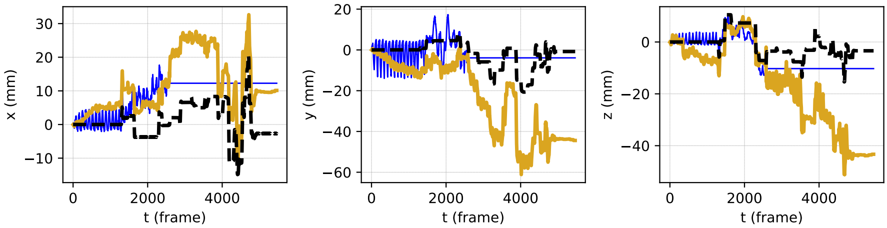

Fig. B6 Trajectory of *StereoMIS* P2_0 (ATE-RMSE 20.5mm).

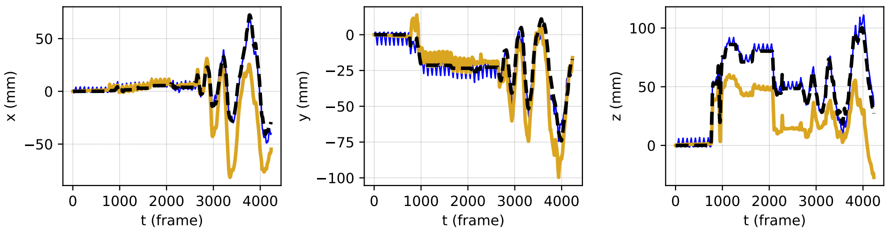

Fig. B7 Trajectory of *StereoMIS* P2_1 (ATE-RMSE 24.2mm).

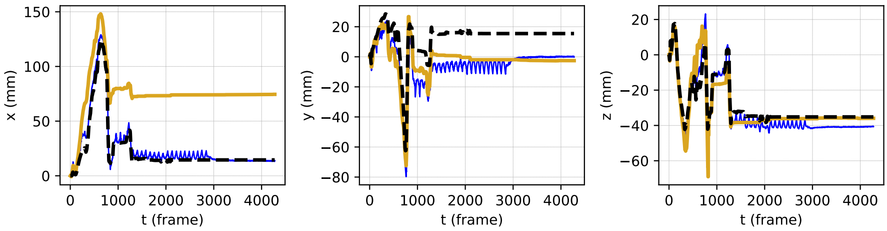

Fig. B8 Trajectory of *StereoMIS* P2_2 (ATE-RMSE 12.8mm).

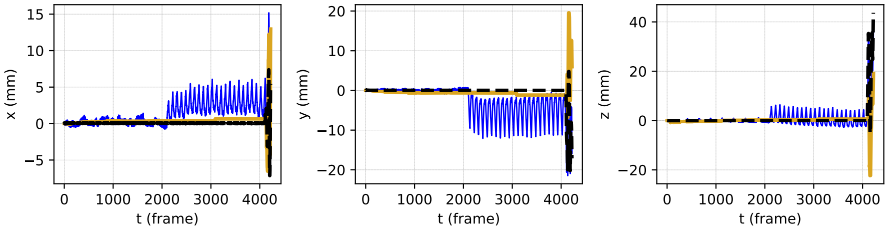

Fig. B9 Trajectory of *StereoMIS* P2_3 (ATE-RMSE 4.3mm).

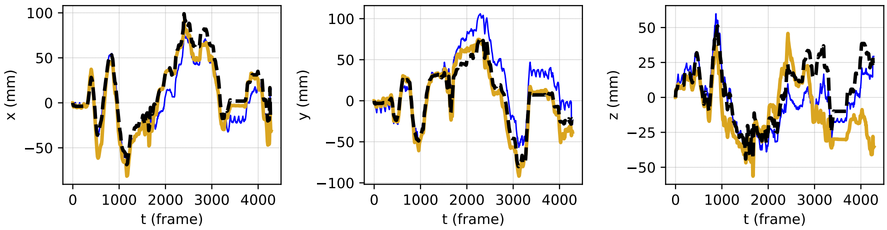

Fig. B10 Trajectory of *StereoMIS* P2_4 (ATE-RMSE 15.1mm).

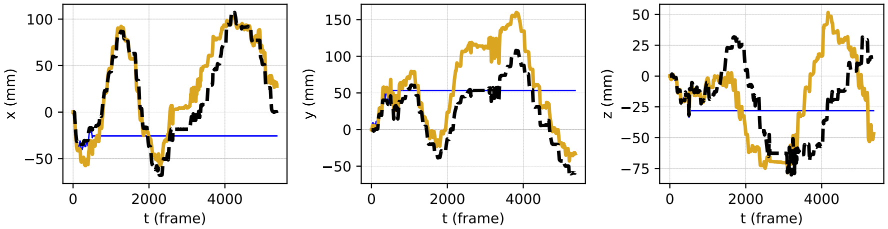

Fig. B11 Trajectory of *StereoMIS* P2_5 (ATE-RMSE 22.7mm).

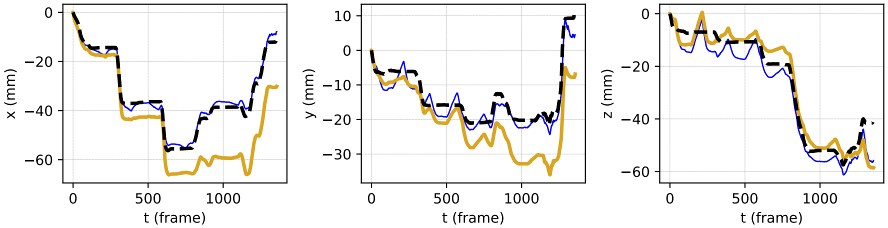

Fig. B12 Trajectory of *StereoMIS* P2_6 (ATE-RMSE 5.4mm).

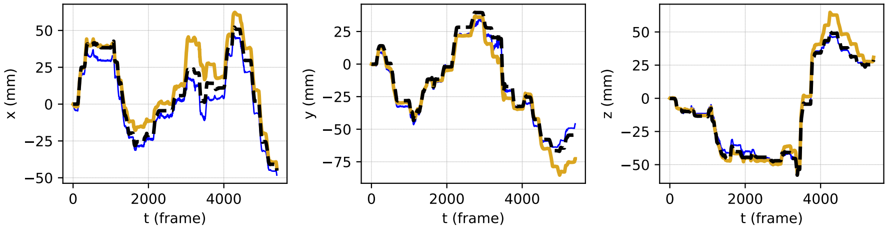

Fig. B13 Trajectory of *StereoMIS* P2_7 (ATE-RMSE 9.3mm).

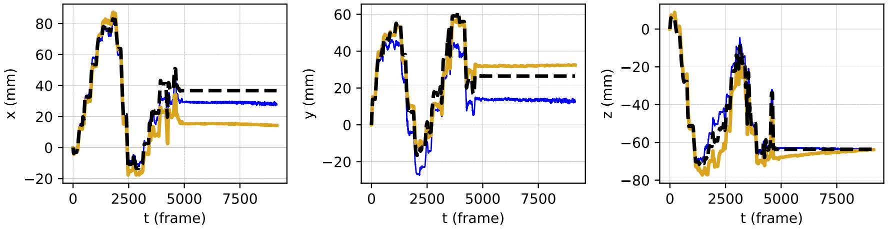

Fig. B14 Trajectory of *StereoMIS* P2_8 (ATE-RMSE 10.2mm).

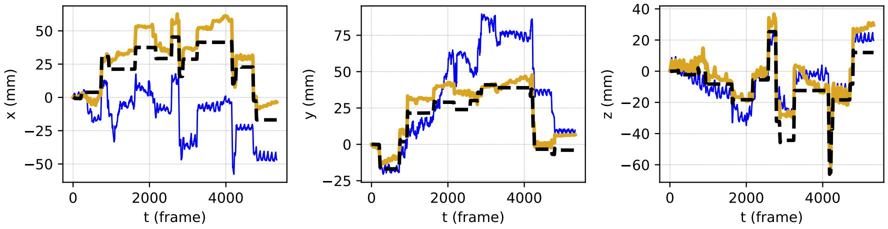

Fig. B15 Trajectory of *StereoMIS* P3_0 (ATE-RMSE 8.8mm).

Appendix C Results on full StereoMIS sequences

Table C2 Pose estimation results on full StereoMIS test sequences for ORB-SLAM2, ElasticFusion, and **ours**. **Frames where SLAM methods fail to estimate the pose are removed from the evaluation**. The success ratio is the ratio of frames where the SLAM method estimates the pose over the total number of frames. Metrics are reported in (mean±std) when applicable.

	H2	H3	P2	P3	macro avg.
			Success Ratio		
ORB-SLAM2	0.34	0.93	0.82	1.0	0.77 ± 0.3
ElasticFusion	0.99	1.0	1.0	1.0	1.0 ± 0.0
**ours**	1.0	1.0	1.0	1.0	1.0 ± 0.0
			ATE-RMSE (mm)		
ORB-SLAM2	14.3	**4.4**	**8.4**	21.4	$\mathbf{12.1 \pm 7.4}$
ElasticFusion	30.8	72.1	32.9	37.7	43.4 ± 19.4
**ours**	**10.9**	21.2	13.8	**8.8**	13.7 ± 5.4
			RPE-trans (mm)		
ORB-SLAM2	0.46 ± 0.53	0.24 ± 0.25	0.40 ± 0.49	0.54 ± 0.47	0.41 ± 0.16
ElasticFusion	0.87 ± 1.11	0.56 ± 1.03	0.83 ± 1.13	0.71 ± 0.79	0.74 ± 0.12
**ours**	$\mathbf{0.10 \pm 0.27}$	$\mathbf{0.10 \pm 0.18}$	$\mathbf{0.16 \pm 0.32}$	$\mathbf{0.19 \pm 0.31}$	$\mathbf{0.14 \pm 0.04}$
			RPE-rot (deg)		
ORB-SLAM2	0.39 ± 0.43	0.16 ± 0.22	0.25 ± 0.34	0.28 ± 0.27	0.27 ± 0.06
ElasticFusion	0.73 ± 1.06	0.41 ± 0.96	0.60 ± 1.15	0.38 ± 0.40	0.53 ± 0.13
**ours**	$\mathbf{0.04 \pm 0.20}$	$\mathbf{0.04 \pm 0.13}$	$\mathbf{0.07 \pm 0.14}$	$\mathbf{0.05 \pm 0.10}$	$\mathbf{0.05 \pm 0.01}$

Appendix D SCARED test set trajectories

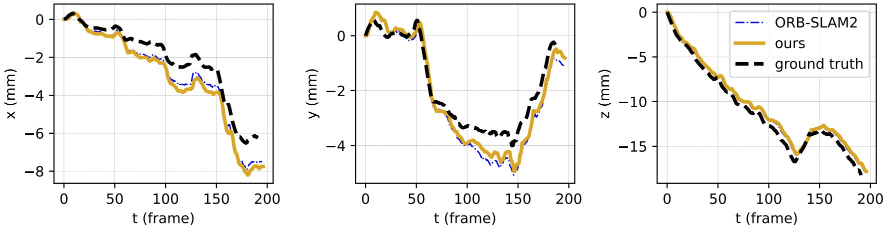

Fig. D16 Trajectory of *SCARED* d1_k1 (ATE-RMSE 0.47mm).

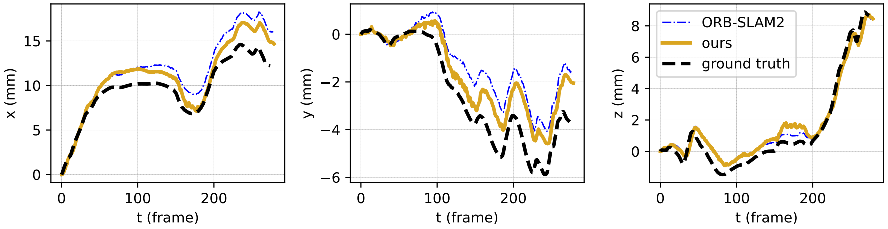

Fig. D17 Trajectory of *SCARED* d1_k2 (ATE-RMSE 0.37mm).

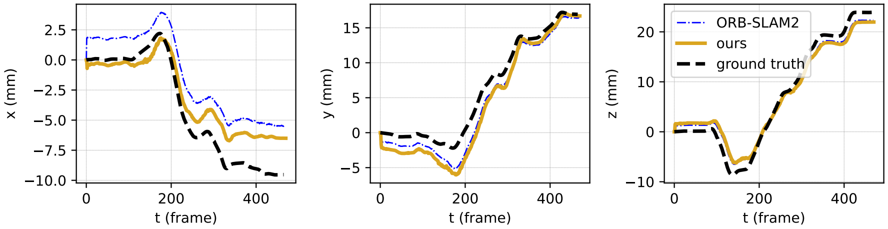

Fig. D18 Trajectory of *SCARED* d1_k3 (ATE-RMSE 1.05mm).

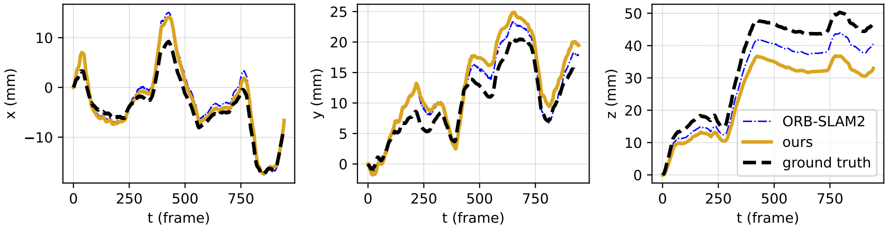

Fig. D19 Trajectory of *SCARED* d8_k0 (ATE-RMSE 3.53mm).

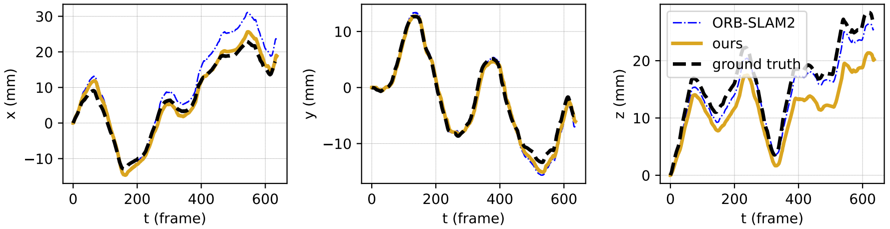

Fig. D20 Trajectory of *SCARED* d8_k1 (ATE-RMSE 2.08mm).

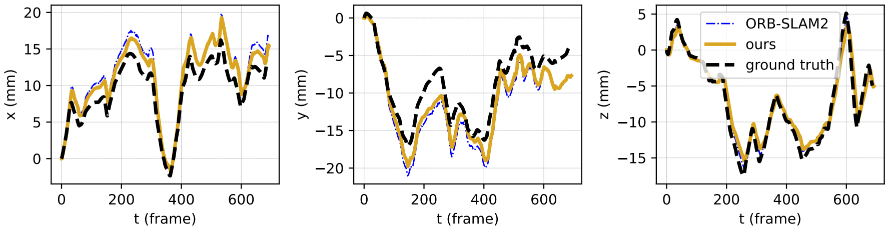

Fig. D21 Trajectory of *SCARED* d8_k2 (ATE-RMSE 1.20mm).

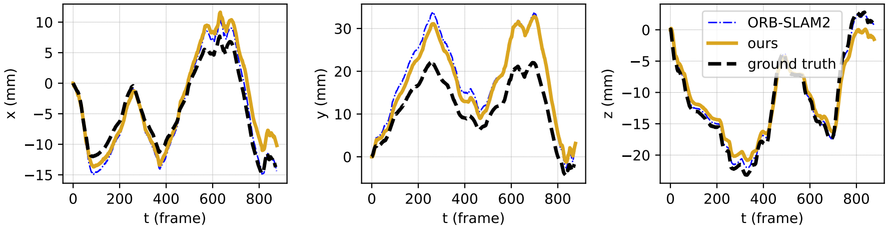

Fig. D22 Trajectory of *SCARED* d8_k3 (ATE-RMSE 1.92mm).

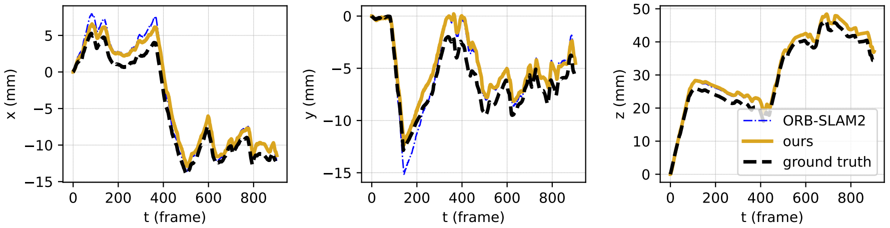

Fig. D23 Trajectory of *SCARED* d9_k0 (ATE-RMSE 0.77mm).

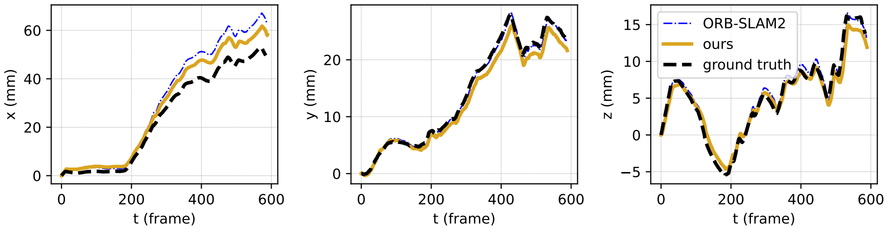

Fig. D24 Trajectory of *SCARED* d9_k1 (ATE-RMSE 2.04mm).

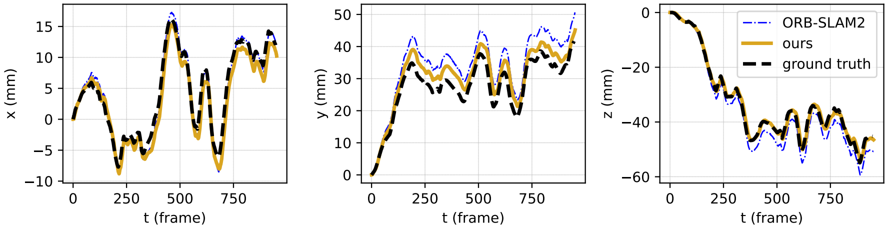

Fig. D25 Trajectory of *SCARED* d9_k2 (ATE-RMSE 0.96mm).

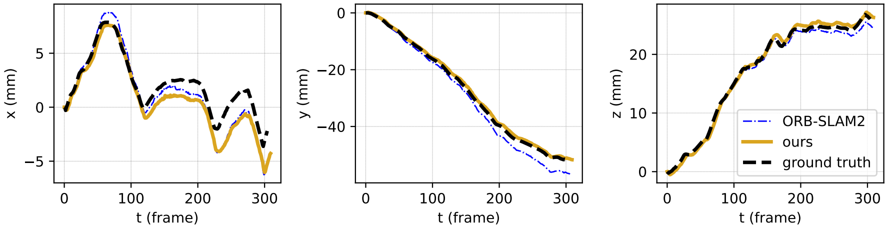

Fig. D26 Trajectory of *SCARED* d9_k3 (ATE-RMSE 0.84mm).
